# The effect of metabolic risk factors on the natural course of gastro-oesophageal reflux disease

**DOI:** 10.1136/gut.2008.162305

**Published:** 2008-10-17

**Authors:** Y-C Lee, A M-F Yen, J J Tai, S-H Chang, J-T Lin, H-M Chiu, H-P Wang, M-S Wu, T H-H Chen

**Affiliations:** 1 Division of Biostatistics, College of Public Health, National Taiwan University, Taipei, Taiwan; 2 Department of Internal Medicine, College of Medicine, National Taiwan University, Taipei, Taiwan; 3 Department of Internal Medicine, E-DA Hospital and I-Shou University, Kaohsiung County, Taiwan

## Abstract

**Background and aims:**

The effect of metabolic risk factors on the natural course of gastro-oesophageal reflux disease (GORD), which remains elusive, was quantified.

**Methods:**

The population included 3669 subjects undergoing repeated upper endoscopy. Data were analysed using a three-state Markov model to estimate transition rates (according to the Los Angeles classification) regarding the natural course of the disease. Individual risk score together with the kinetic curve was derived by identifying significant factors responsible for the net force between progression and regression.

**Results:**

During three consecutive study periods, 12.2, 14.9 and 17.9% of subjects, respectively, progressed from non-erosive to erosive disease, whereas 42.5, 37.3 and 34.6%, respectively, regressed to the non-erosive stage. The annual transition rate from non-erosive to class A–B disease was 0.151 per person year (95% CI 0.136 to 0.165) and from class A–B to C–D was 0.079 per person year (95% CI 0.063 to 0.094). The regression rate from class A–B to non-erosive disease was 0.481 per person year (95% CI 0.425 to 0.536). Class C–D, however, appeared to be an absorbing state when not properly treated. Being male (relative risk (RR) 4.31; 95% CI 3.22 to 5.75), smoking (RR 1.20; 95% CI 1.03 to 1.39) or having metabolic syndrome (RR 1.75; 95% CI 1.29 to 2.38) independently increased the likelihood of progressing from a non-erosive to an erosive stage of disease and/or lowered the likelihood of disease regression. The short-term use of acid suppressants (RR 0.54; 95% CI 0.39 to 0.75) raised the likelihood of regression from erosive to non-erosive disease.

**Conclusions:**

Intraoesophageal damage is a dynamic and migratory process in which the metabolic syndrome is associated with accelerated progression to or attenuated regression from erosive states. These findings have important implications for the design of effective prevention and screening strategies.

Gastro-oesophageal reflux disease (GORD) is becoming increasing prevalent in the population, paralleling similar rises in the frequency of metabolic disorders, and resulting in the concomitant growth of an already considerable economic burden.^1^ Although GORD substantially affects public health, its natural history remains elusive. Two opposing theories have been proposed to explain the GORD heterogeneity. The category theory holds that GORD can be treated as three distinct entities (non-erosive (NE) reflux disease, erosive reflux disease and Barrett’s oesophagus^2^ ^3^) and arises from the fact that therapeutic responses differ substantially between erosive and NE disease stages. The continuum theory suggests that GORD is a spectrum of diseases with differing severities. Support derives from the fact that transitions from NE to erosive disease are observed during endoscopic follow-up,^4^ and disease severity might account for observed variations in therapeutic responses.^5^

Several studies have reported that the adverse effect of obesity on GORD is through mechanical alterations at the oesophagogastric junction.^6^^–^10 However, since not every obese patient develops GORD, the pathogenesis must be multifactorial and cannot be explained by a single physiological parameter.^11^ Therefore, knowledge of GORD’s natural history and its relationship with metabolic risk factors is very informative not only to identify which individuals should undergo endoscopic screening but also to develop individually tailored prevention strategies. Unfortunately, it is difficult to assess GORD’s natural history because of the paucity of data from large, long-term endoscopic follow-up studies and the fact that symptoms of the disease cannot be treated as a surrogate measure for endoscopy. Even when data are available, serial observations with irregular interexamination intervals render the quantification of transition between states and the derivation of kinetic curves that shows how each state evolves with time hard to assess without using complex multistate models. Our primary aim was to quantify the effect of putative factors, particularly the effect of metabolic risk factors, on the rates of onset, progression and regression between NE and erosive disease states.

## METHODS

### Participants and evaluation

Our study was based on a voluntary health promotion programme at National Taiwan University Hospital (NTUH) that used a standard protocol including a physical examination, blood chemistries, plain radiography, abdominal ultrasonography and endoscopy. Most subjects were invited to undergo an upper gastrointestinal endoscopy annually. Such a scheme is confirmed to be effective for cancer prevention in areas where the upper gastrointestinal cancers are prevalent.^12^^–^14 The ethics committee at our hospital approved the study protocol.

We enrolled patients who underwent at least two endoscopic examinations. Excluded were those who received proton pump inhibitors (PPIs) or histamine-2 receptor antagonists (H2RAs) in the 4 months preceding the first endoscopy, those who underwent gastrectomy and those with malignancy. National Health Insurance in Taiwan covers a 4-month course of treatment with a PPI or an H2RA for those who show signs of erosive oesophagitis or peptic ulcer disease after endoscopy.^15^ These medications are not available over-the-counter in Taiwan. Subjects who received less than 4 months of treatment were defined as short-term users. They were included in the analysis since a short-course PPI/H2RA treatment for erosive oesophagitis is the common clinical practice.^16^ Those who required two or more successive courses of PPI or H2RA treatment were defined as long-term users. They were excluded because such treatment may strongly affect GORD’s natural history.

Prior to the examination, a self-administered questionnaire was used to collect information on demographics, social habits and medical/medication histories. We defined symptoms of GORD as the presence of troublesome heartburn, acid regurgitation or both. Heartburn was defined as a burning sensation in the retrosternal area and acid regurgitation as the perception of flow of refluxed gastric contents into the mouth or hypopharynx. The frequency was once a week or more over the past 3 months. Self-reported data were confirmed in a face-to-face interview with an internist.^17^ This GORD-specific approach has been validated in population-scale research.^9^ ^18^

Participants were evaluated for metabolic risk factors, including measurements of body mass index (BMI), waist circumference, blood pressure, plasma glucose, total cholesterol, high-density lipoprotein (HDL) cholesterol, triglycerides and uric acid levels. According to the modified criteria for Asians,^19^ metabolic syndrome is the presence of three or more of the following: waist circumference >90 cm for men or >80 cm for women, serum triglyceride levels ⩾150 mg/dl, HDL cholesterol levels <40 mg/dl, blood pressure ⩾130/85 mm Hg and serum glucose levels ⩾110 mg/dl. Those receiving antidiabetic or antihypertensive therapy were assumed to have high fasting glucose levels or high blood pressure (details have been described elsewhere).^20^

After the evaluation of metabolic risk factors, subjects underwent endoscopy. Erosive oesophagitis was scored using the Los Angeles (LA) classification system with standard comparator photos.^21^ The original LA classification for erosive oesophagitis consists of four classes but we combined these into two categories (classes A–B and C–D) to reduce interobserver variation^22^ and increase statistical power. The overall κ changed from 0.45 to 0.65, suggesting good consistency.^23^

### Three-state Markov model

**Figure 1:**
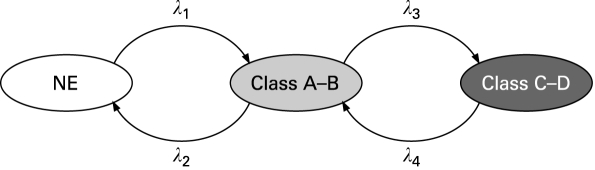
Three-state Markov model of the natural history of gastro-oesophageal reflux disease. The transition rates, λ_1_–λ_4_, are parameters in the model and will be estimated.

We modelled GORD’s natural history as a three-state Markov process by defining state 1 as NE disease, state 2 as LA class A–B oesophagitis and state 3 as class C–D oesophagitis (fig 1). Transitions between serial states were quantified by two instantaneous progression rates from state 1 to 2 (λ_1_) and from state 2 to 3 (λ_3_), and two instantaneous regression rates from state 2 to 1 (λ_2_) and state 3 to 2 (λ_4_).

Cumulative risk for each transition was computed by transition probabilities that were a function of transition rates λ_1_–λ_4_ and follow-up time by using the method of Chen *et al*.^24^ The evolution of these cumulative risk curves corresponding to state-to-state transitions of GORD’s natural course with follow-up time are called kinetic curves. For subjects free of erosive disease at baseline, kinetic curves showed the evolution of cumulative risk for developing class A–B and class C–D oesophagitis and probability of remaining in the NE disease state. For subjects with class A–B disease at baseline, kinetic curves showed the evolution of cumulative risk for developing class C–D, remaining in class A–B and regressing to the NE disease state.

### Statistical analysis

Time intervals between endoscopic examinations were recorded to build up a continuous-time Markov process for the three-state model. We estimated the transition rates labelled in fig 1 and their 95% CIs based on the total likelihood, a product of transition probabilities from a series of endoscopic examinations in all subjects. Statistical analyses for this model have been described in several papers.^24^^–^28 As time intervals between assessments are irregular and vary from individual to individual, a multistate model is adopted to tackle this technical problem with the incorporation of different time intervals into transition probabilities, which is elaborated in Appendix 1.

We presented the model parameters derived from the complete data set of subjects. To test the predictive validity of the current model, we also performed cross-validation by splitting data into 2/3 for deriving the model and 1/3 for validation of the model. The observed transition histories in the validated data set were compared with the predicted ones that were computed by the application of parameters trained from the derived data set.

As we are interested in the effect of metabolic risk factors on transition rates, a univariate regression analysis was therefore done to assess the effect of each component on transition rates. The exponential Markov regression form was adapted to estimate relative risk (RR), which is done by taking exponentials of the regression coefficients of the Markov regression. Besides the metabolic profile, factors considered in the regression included basic demographic information, lifestyle factors, symptoms of reflux and the use of acid suppressants following screening. It should be noted that each predictor in the same individual may vary from time to time and was repeatedly evaluated along with each endoscopic screening. They are treated as time-varying covariates, which means that their contributions to progression and regression of GORD may depend on the status they had at the time preceding the next transition during a given epoch. Thus, the net force of dynamic change of each covariate contributing to progression and regression is worthy of investigation.

Take smoking status (smoking, X = 1; no smoking, X = 0), for example. An individual at time t_0_ was a current smoker (X = 1), he developed A–B during time interval (t_1_–t_0_), quit smoking, was treated as non-smoking (X = 0), at time t_1_, regressed to NE during the time interval (t_2_–t_1_), and stayed as NE without smoking until time t_3_. Thus, as smoking status changed with time, its effect in each epoch makes different contributions to disease progression and regression in the same individual. The net force of smoking on the state-to-state transitions can be considered in this manner to aggregate each individual change into a population-average net effect expressed by the difference of regression coefficients between progression and regression. The same phenomenon may be applied to PPI/H2RA use that is associated with progression on the grounds of indication and regression probably causally related through treatment.

Hypothesis testing for such net force for each risk factor mentioned above is performed as follows. The transition rate function is first developed:

λ*_j_*(*t*) = λ_0*j*_(*t*)exp(β*_j_X*(*t*)), for *j* = 1, 2

where λ_01_ and λ_02_ are baseline progression and regression rates, β_1_ and β_2_ are the corresponding regression coefficients, and *X*(*t*) is the indicator variable concerned with the presence of risk factors at time *t* prior to the next transition. The null hypothesis for an attributed lack of effect due to a risk factor is defined as β_1_–β_2_ (net effect) = 0. The alternative hypothesis was set as β_1_–β_2_≠0, where β_1_–β_2_>0 indicates a detrimental effect and β_1_–β_2_<0 indicates a protection. By using the estimated variance–covariance matrix, the significance of a risk factor was determined using the Wald test statistic.

To build a multivariate model, we used forward selection to evaluate the additive effects of risk factors. The presence of metabolic syndrome, and its individual components, were added one by one into the model. The final model was selected based on the log-likelihood ratio test.^29^ Using the set of risk factors that were significant in the final model, we created a predicted risk score based on regression coefficients estimated from the model and the assigned time-invariant and time-varying factors. Following the above notation of β_1_ and β_2_, the predicted risk score at time *t* (*S*(*t*)) for each set of risk factors is expressed by:


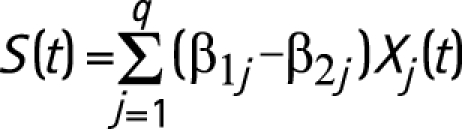


where *X_j_*(*t*) is a set of significant factors and *q* is the number of significant factors. Risk stratification according to this predicted risk score yielded a series of different kinetic curves with different shapes, showing heterogeneous natural courses of disease. Analysis was done using an SAS/IML procedure (version 9.1; SAS Institute Inc., Cary, North Carolina, USA). All p values were two sided; p<0.05 indicated significance.

## RESULTS

### Descriptive findings

Between June 2003 and December 2006, 19 812 subjects underwent screening upper endoscopy at NTUH. Of these, 3669 had at least two examinations and comprised our study group (table 1). Mean age (SD) was 56.3 (10.5) years; mean BMI was 24.1 (3) kg/m^2^. GORD symptoms were reported in 11.3%. The prevalence rate (16.4%) of erosive oesophagitis among these 3669 subjects was similar to that (15.7%) obtained from the whole population (n = 19 812) in the previous study.^30^ This may suggest that we did not have findings on the first endoscopy that warranted more frequent surveillance for these subjects with repeated endoscopy. Stratified by their endoscopic findings, 10.3% of NE, 15.5% of A–B and 35.3% of C–D groups had GORD symptoms. The average duration of short-term PPI or H2RA treatment was 2.1 (0.8) months. Thirty patients (0.15%) were excluded for being long-term PPI or H2RA users. Barrett’s oesophagus was too rare (0.06%) to be included.^30^ As only 0.35% had chronic renal insufficiency (serum creatinine concentration ⩾2 mg/dl), this was also excluded.

**Table 1:** Baseline characteristics of the 3669 study subjects

Characteristic	No. of subjects (%)
Male	2483 (67.7)
Smoker	416 (11.3)
Drinks alcohol (at least once per week)	2219 (60.5)
Chronic illnesses	
Cardiac	47 (1.3)
Pulmonary	92 (2.6)
Hepatic	501 (13.7)
Peptic ulcer	648 (17.7)
Cholesterol ⩾200 mg/dl	314 (8.6)
Hyperuricaemia* and/or history of gout	283 (7.7)
Metabolic syndrome	498 (13.6)
Enlarged waist circumference	1239 (33.8)
Hypertension or blood pressure ⩾130/85 mm Hg	559 (15.2)
Diabetes or fasting glucose ⩾110 mg/dl	198 (5.4)
HDL-C <40 mg/dl	1678 (45.7)
Triglycerides ⩾150 mg/dl	908 (24.7)
Exercise, number of times per week	
⩾5	1013 (27.6)
3–4	1585 (43.2)
⩽2	1071 (29.2)
Sleep quality	
Good	1600 (43.6)
Fair	1564 (42.6)
Poor	505 (13.8)
Symptoms of GORD	413 (11.3)
Short-term use of PPI or H2RA	587 (11.4)†

GORD, gastro-oesophageal reflux disease; H2RA, histamine-2 receptor antagonist; HDL-C, high-density lipoprotein cholesterol; PPI, proton pump inhibitor.

*Serum uric acid concentration >7.5 mg/dl.

†From 5145 transition periods.

### Transition rates between states in GORD’s natural history

Subjects underwent up to four endoscopies creating three epochs (baseline endoscopy to endoscopy 2, endoscopy 2 to 3, and endoscopy 3 to 4). The mean duration of epochs 1, 2 and 3 (in days) was 528 (210), 392 (108) and 352 (60). Table 2 shows aggregate numbers of observed transitions between states.

**Table 2:** Aggregate counts of transitions between states during the three study epochs

Epochs	No. of subjects (%)		
	NE	Class A–B	Class C–D
Baseline	Endoscopy 2 (n = 3669)		
NE (n = 3066)	2693 (87.8)	350 (11.4)	23 (0.8)
Class A–B (n = 586)	249 (42.5)	304 (51.9)	33 (5.6)
Class C–D (n = 17)	0 (0)	0 (0)	17 (100)
Endoscopy 2	Endoscopy 3 (n = 1140)		
NE (n = 930)	791 (85.1)	136 (14.6)	3 (0.3)
Class A–B (n = 198)	74 (37.3)	109 (55.1)	15 (7.6)
Class C–D (n = 12)	0 (0)	0 (0)	12 (100)
Endoscopy 3	Endoscopy 4 (n = 336)		
NE (n = 252)	207 (82.1)	45 (17.9)	0 (0)
Class A–B (n = 78)	27 (34.6)	39 (50)	12 (15.4)
Class C–D (n = 6)	0 (0)	0 (0)	6 (100)

NE, non-erosive state.

There were 5145 transitions, including 3669 in epoch 1, 1401 in epoch 2, and 336 in epoch 3. Observed rates of transition from NE to erosive oesophagitis were 12.2% (95% CI 8.9% to 15.5%), 14.9% (95% CI 9% to 20.8%) and 17.9% (95% CI 13.2% to 22.6%). The risk of progressing from class A–B to class C–D oesophagitis increased from 5.6% (95% CI 3.7% to 7.5%) in epoch 1 to 15.4% (95% CI 7.4% to 23.4%) in epoch 3; the probability of regression from class A–B to NE decreased from 42.5% (95% CI 38.5% to 46.5%) in epoch 1 to 34.6% (95% CI 24.8% to 44.4%) in epoch 3. However, no statistically significant increase in progression or decrease in regression across epochs was noted (p>0.05). Because no class C–D subjects showed regression to class A–B or NE, the annual regression rate from C–D to A–B (ie, λ_4_) was set to zero.

Annual progression rates from NE to A–B (ie, λ_1_) and from A–B to C–D (ie, λ_3_) were 0.151 (95% CI 0.136 to 0.165) and 0.079 (95% CI 0.063 to 0.094) per person year, respectively, and the regression rate from A–B to NE (ie, λ_2_) was 0.481 (95% CI 0.425 to 0.536) per person year. The corresponding figures were 0.139 (95% CI 0.126 to 0.152), 0.084 (95% CI 0.066 to 0.101) and 0.346 (95% CI 0.294 to 0.398) when we excluded the transition histories of being administered with short-term PPI or H2RA treatment preceding the next transition.

### Kinetic curves

**Figure 2:**
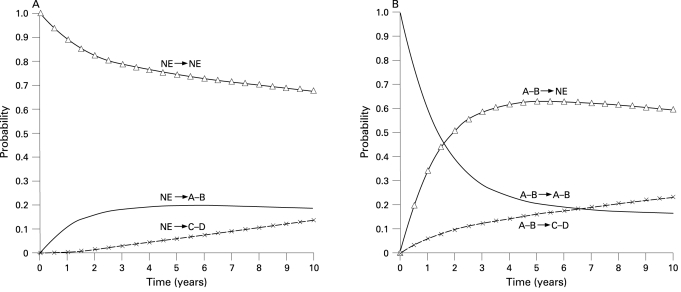
(A) The kinetic curves (cumulative probabilities, see Appendix 2) of transition from non-erosive to class A–B or class C–D disease, and of remaining in the non-erosive state. (B) The kinetic curves (cumulative probabilities) of transition from class A–B to class C–D disease, of remaining in the class A–B disease state, and of regression from class A–B to the non-erosive state.

Model fitting was assessed by comparing predicted with observed transitions using χ^2^; the lack of a significant difference indicated a good fit for the model (p = 0.415). The observed transition histories were still compatible with the predicted values using the cross-validation method (p = 0.876). Figure 2A shows the cumulative risk of developing class A–B (middle curve) and class C–D oesophagitis (bottom curve) and the probability of staying in the NE disease state (upper curve) for subjects free of erosive disease at baseline. After a 10-year follow-up, 68% of patients have NE disease, 19% class A–B, and 13% class C–D. Since disease regression is common, only 8% of patients would stay in the NE state throughout the follow-up period without any transition histories. Figure 2B shows the cumulative risk of progressing to C–D, of remaining in A–B and of regressing to NE for subjects with A–B at baseline, respectively. Of these, 60% would undergo regression to NE within 10 years, 17% would remain as A–B, and 23% would progress to C–D.

### Effects of risk factors on GORD’s natural course

#### Univariate analysis

Table 3 shows the results when we introduced covariates one by one into our model. We only evaluated effects on transitions between NE and A–B because there were too few transitions from A–B to C–D to allow stable parameter estimation.

**Table 3:** Relative risk of transition and corresponding 95% CIs by factors from the univariate three-state Markov model

Variables*	RR (95% CI)†		
	NE→class A–B	Class A–B→NE	Net effect
Age ⩾65 years	1.19 (0.95 to 1.48)	1.02 (0.78 to 1.34)	1.17 (0.91 to 1.50)
Male	2.36 (1.79 to 3.13)‡	0.55 (0.40 to 0.74)‡	4.33 (3.30 to 5.66)‡
Body mass index ⩾27 kg/m^2^	1.28 (1.01 to 1.65)‡	0.70 (0.52 to 0.96)‡	1.81 (1.36 to 2.41)‡
Smoker	2.27 (1.68 to 3.06)‡	1.38 (0.96 to 1.97)	1.65 (1.24 to 2.18)‡
Alcohol use	1.32 (1.08 to 1.62)‡	0.99 (0.78 to 1.26)	1.34 (1.07 to 1.69)‡
Chronic disease			
Cardiac	0.28 (0.07 to 1.16)	1.19 (0.59 to 2.39)	0.23 (0.06 to 1.02)
Pulmonary	0.98 (0.58 to 1.65)	0.53 (0.19 to 1.45)	1.86 (0.72 to 4.80)
Hepatic	1.01 (0.76 to 1.34)	1.09 (0.79 to 1.54)	0.92 (0.67 to 1.26)
Peptic ulcer disease	1.17 (0.92 to 1.49)	0.88 (0.66 to 1.17)	1.33 (1.02 to 1.75)‡
Cholesterol ⩾200 mg/dl	0.86 (0.63 to 1.19)	0.52 (0.34 to 0.79)‡	1.66 (1.08 to 2.55)‡
Hyperuricaemia and/or history of gout	1.46 (1.06 to 1.99)‡	0.90 (0.61 to 1.34)	1.61 (1.12 to 2.33)‡
Metabolic syndrome	1.42 (1.11 to 1.80)‡	0.76 (0.55 to 0.97)‡	1.87 (1.40 to 2.51)‡
Enlarged waist circumference	1.02 (0.84 to 1.25)	0.78 (0.61 to 0.99)‡	1.31 (1.04 to 1.65)‡
Hypertension or blood pressure ⩾130/85 mm Hg	1.32 (1.04 to 1.68)‡	0.87 (0.64 to 1.17)	1.53 (1.15 to 2.04)‡
Diabetes or fasting glucose ⩾110 mg/dl	1.00 (0.68 to 1.48)	0.68 (0.42 to 1.09)	1.48 (0.91 to 2.41)
HDL-C <40 mg/dl	1.39 (1.15 to 1.68)‡	1.00 (0.79 to 1.26)	1.39 (1.12 to 1.73)‡
Triglycerides ⩾150 mg/dl	1.17 (0.95 to 1.45)	0.71 (0.55 to 0.91)‡	1.66 (1.30 to 2.12)‡
Exercise frequency	0.94 (0.76 to 1.17)	1.11 (0.87 to 1.45)	0.84 (0.67 to 1.07)
Sleep quality	1.03 (0.77 to 1.38)	1.12 (0.81 to 1.54)	0.92 (0.68 to 1.25)
Symptoms of GORD	1.23 (0.93 to 1.63)	0.76 (0.54 to 1.05)	1.64 (1.18 to 2.27)‡
Short-term use of PPI or H2RA	1.31 (0.86 to 1.97)	2.83 (2.14 to 3.71)‡	0.46 (0.33 to 0.65)‡

*Factors were dichotomsed (no/yes) as follows: age ⩾65 years, male, body mass index ⩾27 kg/m^2^, smoker, alcohol consumed ⩾ once per week, metabolic syndrome, exercise more than twice per week, poor sleep quality, symptoms of GORD and use of short-term PPI or H2RA. The “no” group constitutes the baseline comparator.

†The RR for evaluating the role of each factor was arrived at by taking the exponential of the regression coefficient (β) of the Markov regression—that is, exp(β_1_) for progression, exp(β_2_) for regression and exp(β_1_–β_2_) for the net effect.

‡p<0.05.

GORD, gastro-oesophageal reflux disease; HDL-C, high-density lipoprotein cholesterol; H2RA, histamine-2 receptor antagonist; PPI, proton pump inhibitor.

Being male (RR = 2.36) and having a BMI ⩾27 kg/m^2^ (RR = 1.28) both raised the likelihood of progressing from NE to erosive disease and lowered the likelihood of regression from erosive to NE disease (RR = 0.55 and 0.70). Smokers and heavy drinkers had a significant risk of erosive disease (RR = 2.27 and 1.32). Subjects with metabolic risk factors, including hypercholesterolaemia, hyperuricaemia, enlarged waist circumference, hypertension, low HDL cholesterol level, hypertriglycaemia and metabolic syndrome, were more likely to progress from NE to erosive disease and/or less likely to regress from erosive to NE states. Short-term PPI or H2RA use increased the likelihood of regression from erosive to NE states (RR = 2.83). GORD symptoms increased the risk of erosive disease (net RR = 1.64).

#### Multivariate analysis

We used forward selection to evaluate the additive effects of covariates on disease onset and regression. The initial model included only gender (the most significant factor in univariate analysis). We then added variables until they stopped adding significantly to the model. The final model included gender, smoking, metabolic syndrome and short-term PPI or H2RA usage (see table 4 for resulting risk estimates).

**Table 4:** Relative risk of transition and corresponding 95% CIs from the multivariate three-state Markov model

Variables*	RR (95%CI)†		
	NE→class A–B	Class A–B→NE	Net effect
Male	2.36 (1.73 to 2.97)‡	0.53 (0.40 to 0.74)‡	4.31 (3.22 to 5.75)‡
Smoker	1.77 (1.32 to 2.36)‡	1.48 (0.98 to 2.15)	1.20 (1.03 to 1.39)‡
Metabolic syndrome	1.29 (1.18 to 1.42)‡	0.74 (0.53 to 0.98)‡	1.75 (1.29 to 2.38)‡
Short-term use of PPI or H2RA	1.73 (0.92 to 2.77)	3.19 (2.32 to 4.44)‡	0.54 (0.39 to 0.75)‡

*Factors were dichotomised (no/yes) as follows: male, smoker, metabolic syndrome and use of short-term use of PPI or H2RA. The “no” group constitutes the baseline comparator.

†The RR for evaluating the role of each factor was arrived at by taking the exponential of the regression coefficient (β) of the Markov regression—that is, exp(β_1_) for progression, exp(β_2_) for regression and exp(β_1_–β_2_) for the net effect.

‡p <0.05.

H2RA, histamine-2 receptor antagonist; PPI, proton pump inhibitor.

The clinical weight each risk factor contributes to (the net effect of regression coefficients) was 1.46 (natural logarithm of 4.31) for male gender, 0.18 for smoking, 0.56 for metabolic syndrome and −0.61 for short-term PPI or H2RA. The predicted risk score at time *t* based on the clinical weight together with risk factor was:

Risk score = (1.46×male)+(0.18×smoking)+(0.56×metabolic syndrome)−(0.61×short-term use of PPI or H2RA)

These dichotomous variables were coded as described in table 4.

**Figure 3:**
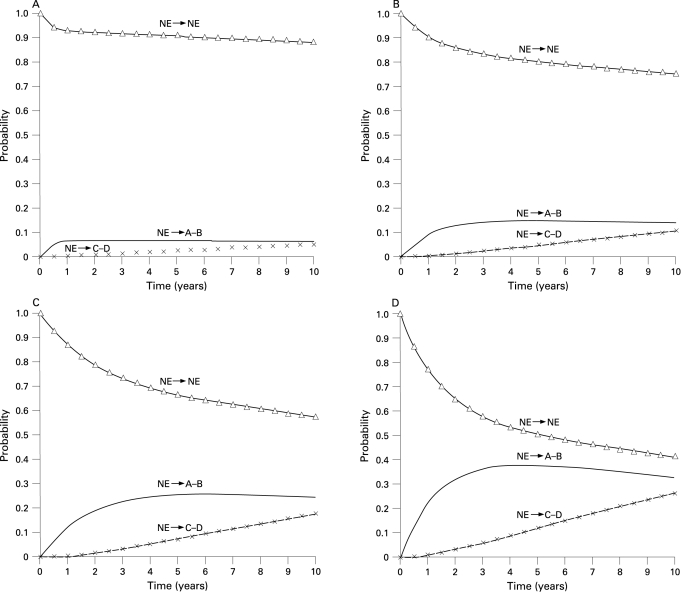
The kinetic curves (cumulative probabilities) of transition from non-erosive to class A–B or class C–D disease, and of remaining in the non-erosive disease state, stratified by individual risk score. A to D illustrate, respectively, the cumulative probability of transition between the above states for those with a risk score of <0, of between 0 and 1, of between 1 and 2, and of >2.

Kinetic curves can be stratified by classifying predicted risk score into four categories, as shown in fig 3A–D. A female with reflux, for example, who does not smoke or have metabolic syndrome but received short-term PPI treatment (total risk score of 0+0+0−0.61 = −0.61), would have the lowest probability of progressing to erosive disease (fig 3A, low risk group). An untreated male smoker with metabolic syndrome (total risk score of 1.46+0.18+0.56−0 = 2.2) would have the highest probability of progressing from NE to erosive disease (fig 3D, high risk group). The higher the risk score, the higher the probability of developing erosive oesophagitis.

## DISCUSSION

We quantified the natural history of GORD by fitting a large longitudinal follow-up database of patients undergoing endoscopy. The step-by-step transitions are a solid demonstration of GORD’s dynamic nature. The predicted risk score may enable clinicians to develop individually tailored preventive strategies.

In addition to cross-validation, several studies support our model’s credibility on external predictive validity. Among patients with NE reflux disease, 5/33 (15%) developed erosive changes within 6 months^31^; 16 of 18 (89%) became erosive 10 years later.^32^ A large database of 2306 GORD patients showed that oesophageal mucosa over 7.6 months was unchanged in 67% of patients (our model predicted 67%), improved in 21% (predicted, 27%) and worsened in 11% (predicted, 6%).^33^ Among 3894 GORD patients followed for 2 years, 25% with NE reflux disease progressed to A–B (predicted, 18%) and 0.6% progressed to C–D (predicted, 1.5%); 37% of those with A–B remained A–B (predicted, 39%) and 61% regressed to NE (predicted, 51%).^4^ Five years later, 72% of those with NE reflux disease at baseline remained NE (predicted, 74%), 22% progressed to A–B (predicted, 20%) and 6% progressed to C–D or Barrett’s oesophagus (predicted, 6%). For patients with class A–B at baseline, 57% regressed to NE (predicted, 63%), 31% stayed A–B (predicted, 21%) and 12% became C–D disease or Barrett’s oesophagus (predicted, 16%).^34^

A plausible link can be established between category and continuum theories. The most significant factor affecting vulnerability to erosive oesophagitis is gender. Hence, a slim female who does not smoke or drink alcohol may remain in the NE state for a long time with little chance of developing erosive disease. An obese male, in contrast, who smokes, drinks heavily and has metabolic syndrome (again a typical picture) would probably progress to erosive disease. The probability of changes in disease status being detected at endoscopy would also increase. Thus, different combinations of risk factors lead to different severities of intraoesophageal damage and the disease appears as a continuum upon endoscopic inspection.

The pathogenesis of reflux symptoms is complicated and cannot be explained solely by intraoesophageal damage. Enhanced peripheral and central neural perceptions of stimuli may be crucial in symptom generation.^35^ As the above illustrates, demographic and endoscopic findings can vary markedly in symptomatic patients and may explain why GORD seems categorical under a symptom-oriented approach. Treating cases of reflux as categorical entities according to the mechanisms of symptom generation is worthwhile; however, the value of identifying risk factors and protecting the oesophageal mucosa from irreversible damage cannot be overemphasised. Since spontaneous regression is still possible in patients with low-grade erosive disease without pharmacological treatment, evaluation of individual risk at this stage would give patients opportunities to modify their behaviour (weight reduction, giving up smoking) and enable clinicians to select patients most likely to develop irreversible changes for endoscopic screening and offer them early pharmacological treatment. This argument has been supported by our findings. After removing the effect of short-term treatment, the regression rate has been attenuated from 0.481 to 0.346. This suggests the short-term treatment effect accounts for 27% (1−0.35/0.48) and other significant risk factors are responsible for 73%. The possibility of disease regression, making allowance for short-term treatment effect, remained and may be related to the modification of other risk factors.

Obesity significantly increased the risk of GORD symptoms, erosive oesophagitis, Barrett’s oesophagus and oesophageal adenocarcinoma.^6^^–^8 However, obesity cannot be the sole reason. Hypertension was found, after adjusting for BMI, to be associated with erosive oesophagitis.^36^ In a Japanese population, male sex, obesity, hyperglycaemia and hypertension were independent risk factors for erosive oesophagitis.^37^ Investigating a database of a Korean population, the presence of metabolic syndrome and a higher visceral adipose tissue area were risk factors.^38^ Consistent with the above, we found an association between several metabolic risk factors and accelerated progression to or attenuated regression from erosive disease, which suggests a potential benefit of treating metabolic disorders in GORD patients. The recommendation to abstain from smoking and alcohol is justified.

Our results are credible for several reasons. First, we had numerous cases of NE and were able to assess progression to erosive disease. The simultaneous evaluation of symptomatic and asymptomatic subjects also enabled us to observe the entire disease spectrum. Secondly, all our endoscopists completed the same training programme using a standardised rating protocol. This substantially reduced heterogeneity amongst observers and strengthened our ability to model natural history. Thirdly, we found no spontaneous regressions from high-grade erosive states, which conflicts with findings that 42% of patients with class C–D disease regress to A–B and 50% to NE disease within 2 years.^4^ These latter results were obtained from symptomatic patients with GORD who were participating in a therapeutic trial and are potentially confounded by pharmacological effects. The fact that most of our participants remained asymptomatic, even in high-grade erosive states, lowered their incentive to seek treatment and allowed us to assess the uninterrupted natural history of the disease.

Because progression of GORD is orderly, the Markov approach was appropriate for modelling. However, our target group tended to reflect the general population, so there were few transitions from low- to high-grade oesophagitis and we were unable to investigate the effects of covariates on this stage. Secondly, information about *H pylori* infection was not available. For subjects with antrum-predominant gastritis, *H pylori* may increase gastric acid secretion and thus increase the risk of GORD.^39^ ^40^ This is supported by our finding that peptic ulcer disease (mostly duodenal ulcer) at baseline was associated with a higher risk of progression to an erosive state. However, the effect of *H pylori* eradication on GORD should be investigated. Finally, a short course of acid-suppressing treatment is common among patients with minor erosive disease, and considering this factor in the Markov model may improve its ability to predict disease progression.^16^ However, the exclusion of long-term users of PPIs or H2RAs may limit the generalisability of the model to patients with earlier onset disease or more severe disease. The evaluation of a large and longitudinal database is warranted in order to update our parameter estimates and extend our model to include patients who require long-term treatment with acid-suppressing medication and who are diagnosed with Barrett’s oesophagus.

Our findings suggest that intraoesophageal damage develops as a dynamic process over time. Risk factors in susceptible individuals modulate the likelihood of state-to-state transitions, resulting (upon endoscopy) in the appearance of a continuous spectrum of disease. GORD can therefore be staged with respect to the extent of progression, as with many other chronic diseases. The translation of the quantified knowledge of GORD’s natural history into predicted risk score together with the kinetic curve will be vital for developing individually tailored prevention and screening programmes, to identify candidates for potential interventions, and to determine optimal timing of proposed interventions.

## Appendix 1

### Model specification and the likelihood function for data on endoscopic examination

The transition rates in our three-state model can be expressed in an intensity matrix as follows:

where states 1, 2 and 3 represent states NE, LA class A–B and LA class C–D, respectively. The four transition parameters of λ_1_–λ_4_ have been defined in the text and fig 1. Using the Kolmogorov equation,^25^ the corresponding transition probabilities can be expressed as follows:

where *P_ij_*(*t*)  =  *P*r{X(*t_l_*)  =  *j*|*X*(*t_l–1_*)  =  *i*} is a function of λ_1_–λ_4_, 0 <*t_l–1_* < *t_l_*; *i*, *j* = 1, 2, 3.

Each individual has his/her own history of endoscopic examination. The likelihood function is established based on the transition probabilities with the use of the data on the history of examinations, in order to estimate the transition parameters λ_1_–λ_4_. Such a type of data could be, for example, a man of age 53 years who had undergone repeated endoscopic examinations with the results as follows: NE with smoking on 12 September 2003, class A–B non-smoking on 27 September 2004, NE on 22 July 2005, and NE, again, on 1 June 2006. According to the “lack memory” property of a Markov process, this personal history can be decomposed into three epochs according to the consecutive four endoscopic examinations: (NE→class A–B, 1.04 years| smoking on 12 September), (class A–B→NE, 0.82 years| non-smoking on 27 September), (NE→NE, 0.86 years| still non-smoking on 22 July), as shown for the study group as a whole in table 2. The likelihood of an individual following this history is *P*_12_(1.04| smoking)× *P*_21_(0.82| non-smoking)× *P*_11_(0.86| non-smoking). Note that irregular times are specified for different individuals. To generalise, if *n* individuals are assessed at times *t*_0_, *t*_1_, *t*_2_ and *t*_3_, creating three epochs (*t*_1_–*t*_0_), (*t*_2_–*t*_1_) and (*t*_3_–*t*_2_), the total likelihood function for all individuals can be written as:

where the *n_ijl_* denotes the number of individuals who are in state *i* at time *t_l_*_–1_ and in state *j* at time *t*_l_. The maximum likelihood estimates of the transition rates can be obtained using the Newton–Raphson procedure.

## Appendix 2

### Kinetic curves based on the estimated transition rates

Given the estimated annual progression rates from NE to class A–B oesophagitis and from A–B to class C–D oesophagitis of 0.151 (λ_1_) and 0.079 (λ_3_) per person year, respectively, and the regression rate from class A–B to NE and from C–D to class A–B of 0.481 (λ_2_) per person year and 0 (λ_4_), respectively, the transition rate matrix can be expressed as follows:

The cumulative risk (probability) over time can be calculated using an SAS/IML procedure.^26^ For example, the first year cumulative risk is demonstrated as follows:

where the upper and middle rows indicate the first year cumulative risks depicted in the kinetic curve 2A and 2B, respectively.
